# Covalent protein display on Hepatitis B core-like particles in plants through the in vivo use of the SpyTag/SpyCatcher system

**DOI:** 10.1038/s41598-020-74105-w

**Published:** 2020-10-13

**Authors:** Hadrien Peyret, Daniel Ponndorf, Yulia Meshcheriakova, Jake Richardson, George P. Lomonossoff

**Affiliations:** 1 Department of Biological Chemistry, John Innes Centre, Norwich, NR4 7UH UK; 2 Department of Cell and Developmental Biology, John Innes Centre, Norwich, NR4 7UH UK

**Keywords:** Proteins, Molecular engineering, Molecular biology, Molecular engineering, Nanobiotechnology, Biotechnology, Molecular engineering in plants

## Abstract

Virus-like particles (VLPs) can be used as nano-carriers and antigen-display systems in vaccine development and therapeutic applications. Conjugation of peptides or whole proteins to VLPs can be achieved using different methods such as the SpyTag/SpyCatcher system. Here we investigate the conjugation of tandem Hepatitis B core (tHBcAg) VLPs and the model antigen GFP in vivo in *Nicotiana benthamiana*. We show that tHBcAg VLPs could be successfully conjugated with GFP in the cytosol and ER without altering VLP formation or GFP fluorescence. Conjugation in the cytosol was more efficient when SpyCatcher was displayed on tHBcAg VLPs instead of being fused to GFP. This effect was even more obvious in the ER, showing that it is optimal to display SpyCatcher on the tHBcAg VLPs and SpyTag on the binding partner. To test transferability of the GFP results to other antigens, we successfully conjugated tHBcAg VLPs to the HIV capsid protein P24 in the cytosol. This work presents an efficient strategy which can lead to time and cost saving post-translational, covalent conjugation of recombinant proteins in plants.

## Introduction

Virus-like particles (VLPs) are protein complexes that are similar or even indistinguishable from native viral particles in terms of their structure and antigenicity. They do not contain the viral genome and hence, they cannot replicate and are not pathogenic. The repetitive surface structure and a typical size range of 20–200 nm make VLPs highly immunogenic and they can induce a strong humoral and cellular immune response^1^. They can be utilized as vaccines against the virus they are derived from or can be used as nano-carriers displaying foreign antigens or cell targeting ligands for vaccine or therapeutic approaches (as reviewed in^2–4^).

VLPs derived from Hepatitis B virus (HBV) core protein (HBcAg) are well characterized and extensively studied as antigen-display systems in vaccine design^5–10^ and cancer therapy^11,12^. The HBcAg self-assembles into icosahedral VLPs composed of dimers of the core protein^13^. These VLPs (sometimes referred to as core-like particles, or CLPs) are highly immunogenic and adjuvantic and can be used to present foreign peptides or epitopes in the major immunogenic region (MIR) and at the *N*- or *C*-terminus of HBcAg^14,15^. Depending on the site of integration, the foreign epitope can be presented on the inside of the VLPs (fused to the *C*-terminus), on the surface within the canyons surrounding the spike (integrated at the *N*-terminus), or on the most exposed tip of the spike in the c/e1 loop in the MIR. Furthermore, HBcAg VLPs can be loaded with toll-like receptor ligands enabling further improvements of immunogenicity^16–19^. However, the genetic integration of foreign epitopes into the core protein is limited in terms of the length and structure of the insert which can be incorporated before negatively influencing VLP assembly, stability, solubility as well as correct antigen folding^20–22^. While different strategies, including mosaic particles^21,23,24^, the split core^25–27^ and tandem core^28^ technology, have been developed to allow insertion of larger and structurally more complex proteins, steric hindrance is still a limiting factor. An alternative that can avoid this is chemical conjugation^29^. This strategy has been successfully used to conjugate HBcAg VLPs with bacterial flagellin^30^ and Jegerlehner et al.^31^ showed that introduction of a lysine in the MIR region enables chemical conjugation of HBcAg VLPs and a FLAG model peptide containing a *N*-terminal cysteine. The drawback of chemical conjugation is that it requires the separate expression and purification of the conjugation partners, followed by an in vitro reaction and another purification step to separate the product from residual reactants. Moreover, it is not always possible to fully control the site of conjugation on the VLP or the occupancy of displayed protein. Chemical conjugation can therefore be more time- and cost-intensive compared to genetic fusion^29^. Furthermore, chemical integration does not necessarily lead to a unidirectional antigen display on the surface, which might induce a more efficient immune response compared to multi-directional display^32^. Unidirectional display of antigens avoiding steric hindrance can be achieved using the SpyTag/SpyCatcher technology. This system is based on the affinity of reactive groups identified in the CnaB2 domain of the fibronectin-binding protein, FbaB, from *Streptococcus pyogenes*^33,34^. SpyTag is a 13 amino acid—long peptide of which the first 10 amino acids interact with SpyCatcher^35^ and contains aspartic acid as one reactive group. SpyCatcher is a protein with a size of about 15 kDa containing a reactive lysine group and a catalytic glutamic acid^33^. In the presence of each other, the carboxyl group of the reactive aspartic acid on SpyTag and the ε-amino group of the lysine of SpyCatcher form a covalent isopeptide bond^35–37^. The conjugation reaction can be induced in vitro by mixing two purified components or in vivo by co-expression of both conjugation partners in the same cell^33^. The SpyTag/SpyCatcher strategy has been successfully used to conjugate different antigens to VLPs derived from bacteriophage AP205^38^, HBV surface antigen^39^, potato virus X^40^ or lentiviruses^41^. It has been employed as part of various vaccine design strategies with growing success^42,43^.

For the first time, we analysed the potential of combining the advantages of genetic integration and the SpyTag/SpyCatcher system in plants. While the SpyTag/SpyCatcher system might enable improved antigen display, the in vivo conjugation in plants might offer a highly scalable, cheap and safe way for recombinant protein expression, avoiding in vitro conjugation and unnecessary purification steps. For this, we integrated either the SpyTag or SpyCatcher into tandem HBcAg (tHBcAg) VLPs^28^, the green fluorescence protein (GFP) or the HIV capsid protein P24. The tHBcAg construct consists of tandem repeats of the HBcAg protein genetically fused together via a flexible linker, so as to produce the HBcAg dimer as a single polypeptide chain. This allows just one of the two MIR loops in each dimer to be modified with a protein insert, thereby reducing steric hindrance on the dimer structure^28^. GFP was chosen because it has previously been used as a model antigen for HBcAg VLP display strategies^28,44^, while P24 served as a test viral antigen to demonstrate the applicability of the GFP results. All recombinant proteins where expressed in the cytosol of *Nicotiana benthamiana* using the pEAQ-*HT* expression system^45^. To investigate the possibility of using this technology to display glycosylated proteins, we also localized the conjugation reaction of tHBcAg and GFP to the endoplasmic reticulum (ER).

The results showed that in vivo conjugation of tHBcAg VLPs in both the cytosol and ER of *N. benthamiana* is possible. This work represents an important step to achieve antigen display in plants with limited expression and purifications steps.

## Results

**Figure 1:**
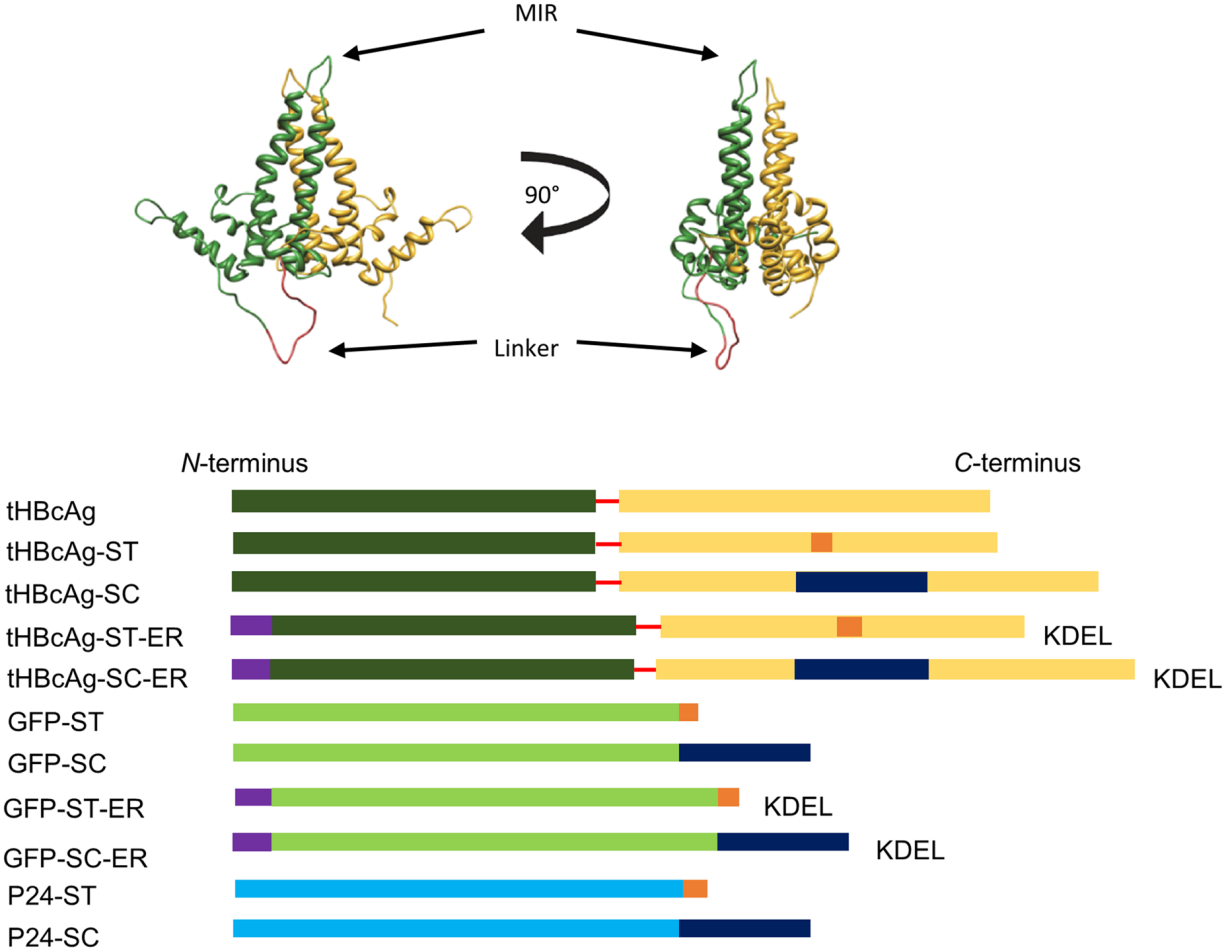
Tandem core technology and constructs used in this study: Top: Structure of tandem core protein as published by Peyret et al.^28^. *N*-terminal core 1 (in dark green) is fused via a flexible linker (red) to *C*-terminal core 2 (yellow). *MIR* major immunogenic region. Bottom: The sequence of SpyTag (ST, orange) or SpyCatcher (SC, dark blue) was inserted into the MIR of the *C*-terminal core 2 of tHBcAg for exposed display on the spike, and the *C*-terminus of GFP (light green) and P24 (teal) respectively. For localization to the endoplasmic reticulum (ER) the transit peptide of *Arabidopsis thaliana* basic chitinase (purple) was fused to the N-terminus of GFP and tHBcAg. For ER retention a *C*-terminal KDEL sequence was added to GFP and tHBcAg.

We designed different constructs to analyse if we could directly decorate tHBcAg VLPs with GFP and P24 using the SpyTag/SpyCatcher (ST/SC) system (Fig. 1). The sequence of either ST or SC was integrated into the MIR of the C-terminal copy of HBcAg region on the tHBcAg protein construct (tEL as described in Peyret et al.^28^), to display the reactive groups on the most exposed region of the VLPs, and the *C*-terminus of GFP and P24, respectively. All four resulting constructs were either expressed in the cytosol or targeted to the ER using an *N*-terminal *Arabidopsis thaliana* basic chitinase ER transit peptide and a *C*-terminal KDEL for ER retention. Successful targeting of the ST/SC system to the ER might ultimately enable in vivo conjugation of VLPs and glycosylated antigens also targeted to the ER. All constructs, including pEAQ-*HT* as empty vector control, were expressed in three week- old *Nicotiana benthamiana* plants. After infiltration, the infiltrated leaf phenotype was monitored and double-layer sucrose cushions^46^ were used to separate VLPs from unconjugated protein. The conjugation reaction and VLP assembly were analysed using Western blotting and transmission electron microscopy (TEM).

### The phenotype of infiltrated leaves indicates the success of the conjugation reaction

**Figure 2:**
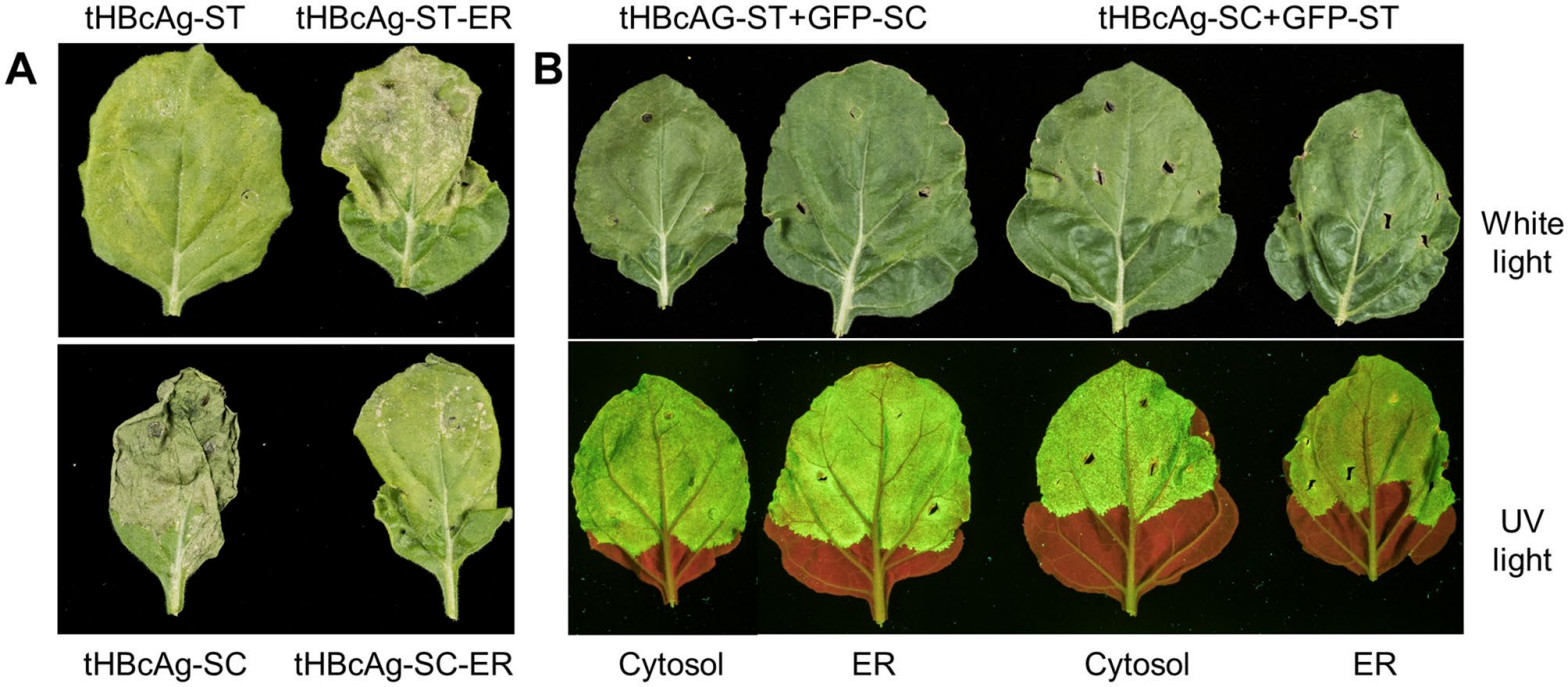
Phenotypes of leaves expressing tandem Hepatitis B core antigen—SpyTag/Catcher (tHBcAg-ST/SC) with or without co-expression of GFP-ST/SC in the cytosol and the endoplasmic reticulum (ER). (**A**) Expression of tHBcAg-ST/SC monitored under white light only. (**B**) Co-expression with GFP-ST/SC monitored under white light and UV-light.

The expression of tHBcAg-ST caused severe necrosis when expressed in the ER, but not in the cytosol. In contrast, tHBcAg-SC caused necrosis when expressed in both compartments (Fig. 2a), but this was more severe in the cytosol. However, the necrotic effects completely vanished when these constructs were co-expressed with their respective GFP conjugation partners (Fig. 2b). Note that leaves expressing “empty loop” untagged tHBcAg^28^ or GFP conjugated to either SpyTag or SpyCatcher do not exhibit any necrosis (see Supplementary Fig. S1). This might indicate that the reactive groups of SpyTag and SpyCatcher on the VLP surface interact spuriously with endogenous plant proteins causing adventitious coupling onto particles with high avidity, resulting in protein cross-linking and ultimately, the observed necrosis. In the presence of the conjugation partner, the binding affinity of SpyTag to SpyCatcher (and vice versa) is likely to be more efficient than these spurious interactions with endogenous proteins, mitigating non-specific cross-binding and the necrotic effect. Fluorescence analysis of infiltrated leaves confirmed that GFP expression occurred to a comparable extent in all groups, showing that fusion to SpyTag or SpyCatcher does not prevent proper folding of GFP (Fig. 2b).

### Conjugation of tHBcAg and GFP in the cytosol

**Figure 3:**
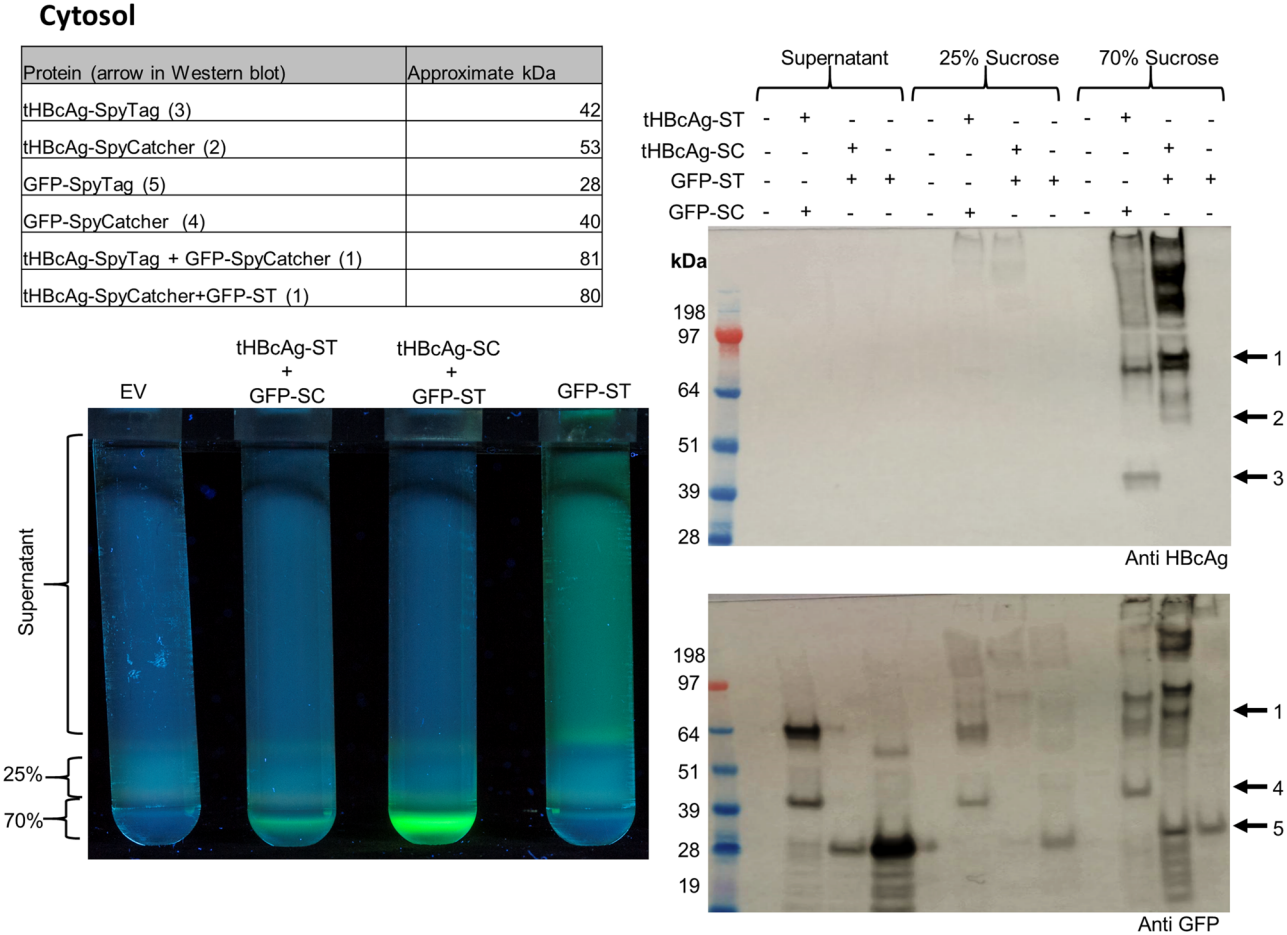
In vivo conjugation of GFP-ST/SC to tHBcAg-SC/ST VLPs in the cytosol: The table in the top left indicates the approximate expected sizes of conjugated and unconjugated proteins. Bottom left: Fluorescence imaging of 25 and 70% double sucrose cushion. *EV* pEAQ-*HT* empty vector control. Unconjugated GFP-ST was mainly detected in the supernatant with some fluorescence in the interface between the 25 and 70% fraction. In the presence of tHBcAg, GFP fluorescence was mainly detected in the 70% sucrose fraction indicating conjugation of VLPs and GFP. Right: Western blot (anti HBcAg = top, anti GFP = bottom). Empty pEAQ-HT vector was used as negative control. Arrow 1 = approximate size of conjugated tHBcAg and GFP (~ 80 kDa). Arrow 2 = unconjugated tHBcAg-SC (~ 53 kDa), arrow 3 = unconjugated tHBcAg-ST (~ 42 kDa), arrow 4 = unconjugated GFP-SC (40 kDa), arrow 5 unconjugated GFP-ST (28 kDa). Unconjugated GFP was mainly detected in the supernatant while tHBcAg was detected in the 70% sucrose fraction. Presence of ~ 80 kDa bands (arrow 1) in 70% fraction in both Western blots indicated successful conjugation of GFP and VLPs. Negative control (EV) shows that all bands are specific for HBcAg or GFP respectively. Blots cropped for clarity, for uncropped blots see Supplementary Fig. S2.

To analyse conjugation of tHBcAg VLPs and GFP, infiltrated leaves were harvested 7 days post-infiltration (dpi) and VLPs were separated from unconjugated GFP by centrifugation over a double-layer sucrose cushion. Viewing the gradients under UV light after ultracentrifugation showed separation between GFP-ST, which was mainly present in the supernatant, and GFP conjugated to tHBcAg VLPs, which was mainly present in the 70% fraction (Fig. 3). This was observed for both combinations of ST and SC and indicated that the GFP had been successfully coupled to tHBcAg. Western blot analysis of the 70% and 25% sucrose fraction as well as the supernatant (Fig. 3) confirmed the conjugation of tHBcAg-ST/SC and GFP-SC/ST by the presence of 80 kDa bands which represents the size of covalently conjugated proteins. However, unconjugated tHBcAg and GFP were observed as well, indicating that GFP occupancy is below 100%. The intensity of fluorescence and the signal in the Western blot of the 70% sucrose fractions also showed that the combination of tHBcAg-SC and GFP-ST seems to be more efficient than tHBcAg-ST and GFP-SC at creating the covalent linkage. This effect was consistent in replicates of this experiment.

### Conjugation of tHBcAg and GFP in the ER

**Figure 4:**
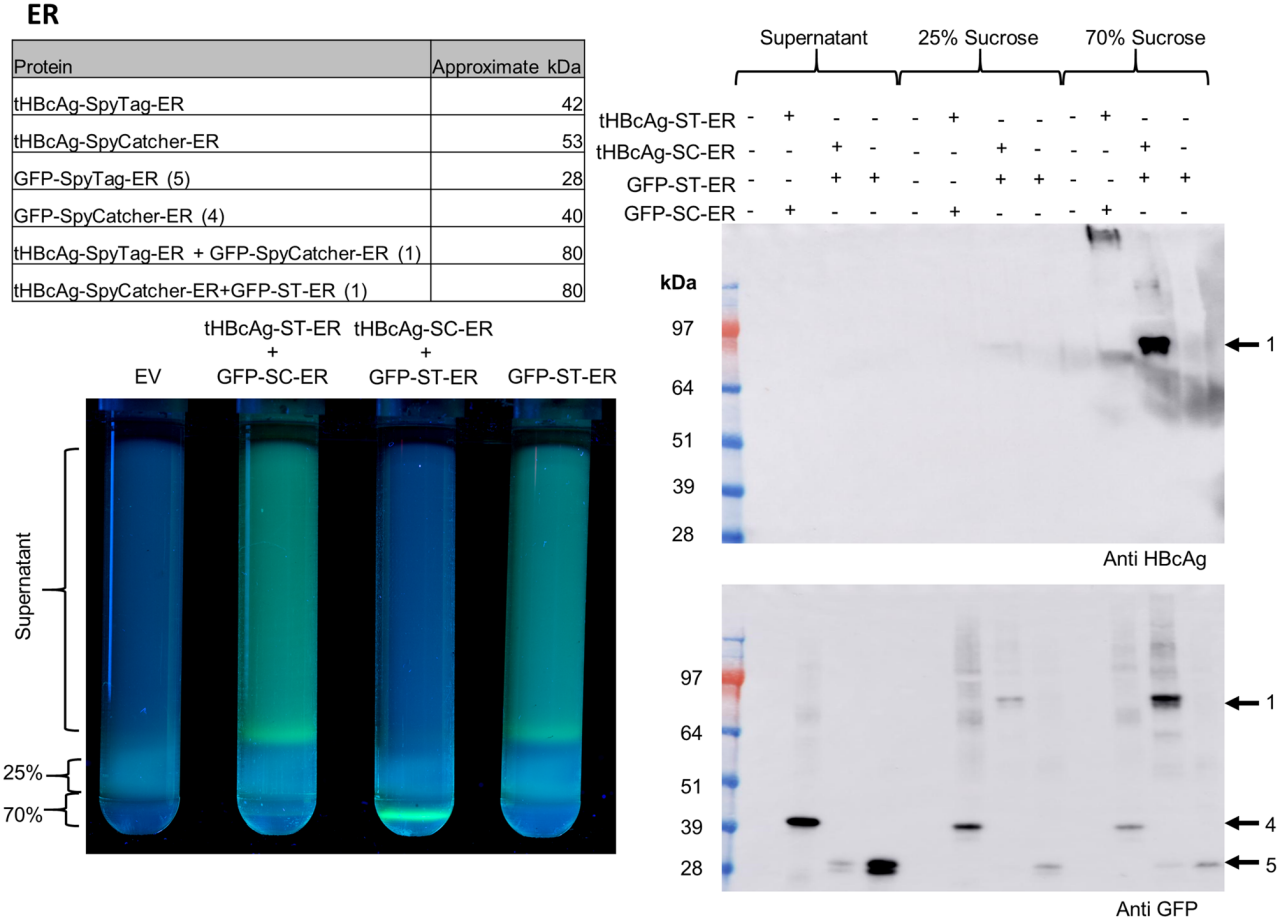
In vivo conjugation of GFP-ST/SC to tHBcAg-SC/ST VLPs in the endoplasmic reticulum (ER): The table in the top left indicates the approximate expected sizes of conjugated and unconjugated proteins. Bottom left: Fluorescence imaging of 25 and 70% double sucrose cushion. pEAQ-*HT* = empty vector control. Unconjugated GFP-ST-ER was mainly detected in the supernatant with some fluorescence in the interface between the 25 and 70% fraction. In presence of tHBcAg-SC-ER VLPs fluorescence was mainly detected in the 70% sucrose fraction indicating conjugation of VLPs and GFP-ST-ER. In contrast tHBcAg-ST-ER and GFP-SC-ER did not seem to result in conjugated VLPs. Right: Western blot (anti HBcAg = top, anti GFP = bottom). Arrow 1 = approximate size of conjugated tHBcAg-ST/SC-ER and GFP-ST/SC-ER (~ 80 kDa), arrow 4 = unconjugated GFP-SC-ER (40 kDa), arrow 5 unconjugated GFP-ST-ER (28 kDa). Unconjugated GFP-ST/SC-ER was mainly detected in the supernatant while tHBcAg-ER was detected in the 70% sucrose fraction. Presence of ~ 80 kDa bands (arrow 1) in 70% fraction in both Western blots indicated successful conjugation of GFP and VLPs. No unconjugated tHBcAg-ST/SC-ER was detected. GFP-SC-ER seems to be mostly unconjugated. Negative control shows that all bands are specific for HBcAg or GFP respectively. Blots cropped for clarity, for uncropped blots see Supplementary Fig. S3.

Targeting of tHBcAg-ST/SC and GFP-SC/ST to the ER led to similar results as cytosolic expression. After sucrose cushion separation, UV-light visualisation of the gradients (Fig. 4) showed that GFP-ST-ER is mainly detected in the supernatant, while tHBcAg-SC-ER co-expressed with GFP-ST-ER led to presence of GFP fluorescence in the 70% fraction, indicating conjugation of GFP and VLPs. This was confirmed by Western blot analysis where GFP in the conjugated size of about 80 kDa could be detected in the 25% and 70% fractions (Fig. 4).

However, in contrast to the conjugation in the cytosol, there was little clear evidence of conjugation between tHBcAg-ST-ER and GFP-SC-ER within the ER (Fig. 4). Fluorescence analysis of the sucrose cushion shows that the majority of GFP-SC-ER is present in the supernatant despite the co-expression of tHBcAg-ST-ER. This observation was confirmed in “Western blot analysis” section where the majority of GFP-SC-ER was detected in the unconjugated size.

### Covalent binding is caused by the SpyTag/SpyCatcher interaction

**Figure 5:**
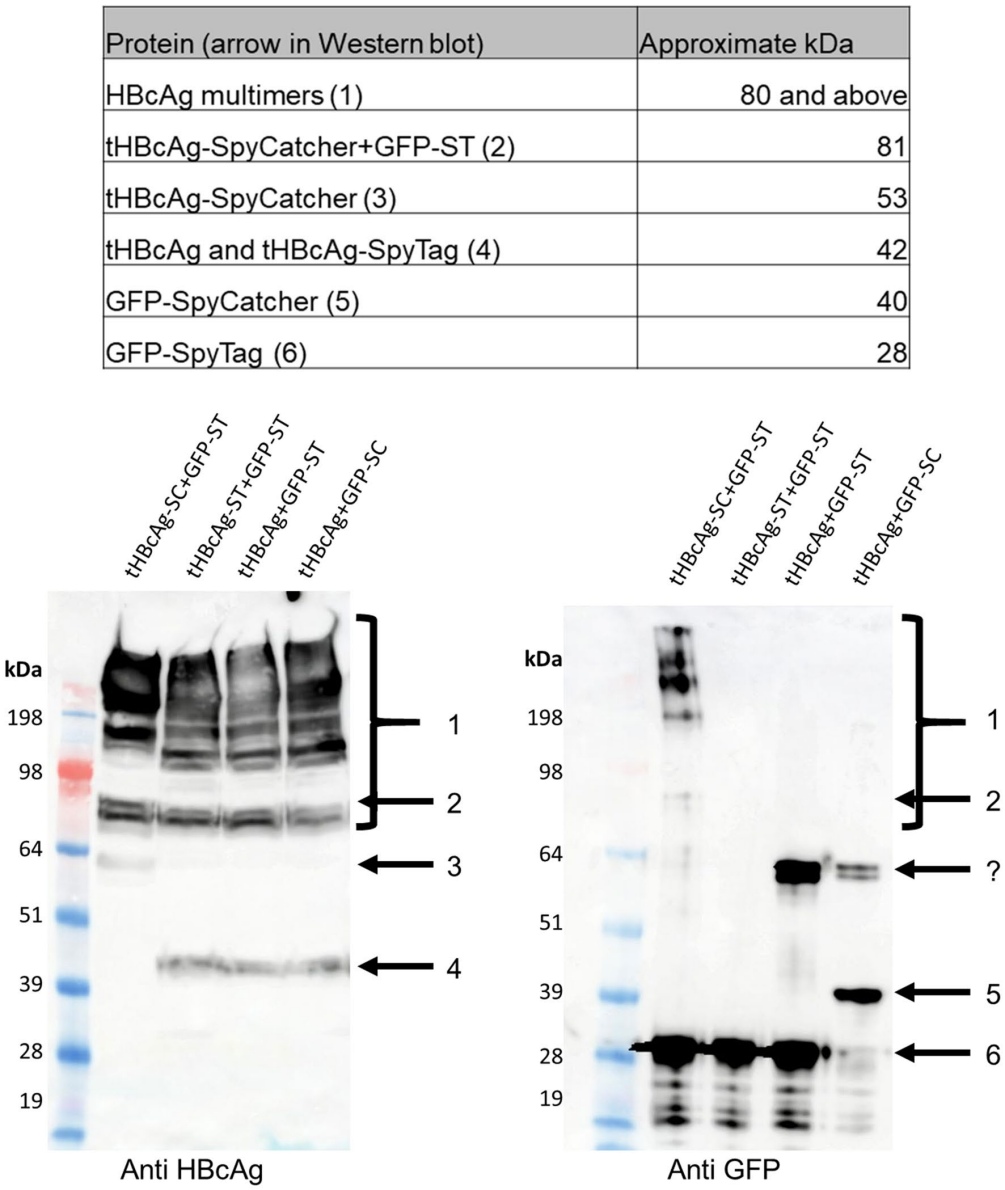
Covalent binding is caused by interaction of SpyCatcher with SpyTag. The table at the top indicates the approximate expected sizes of conjugated and unconjugated proteins. Bottom: duplicate anti-HBcAg (left) and anti-GFP (right) western blots of total soluble protein obtained from leaves co-expressing the proteins indicated. Higher molecular weight multimers of tandem core proteins are visible in the anti-HBcAg blot for all constructs, but these are only visible in the anti-GFP blot when the SpyCatcher/SpyTag interaction can take place (first lane), indicating covalent binding of GFP to HBcAg. Monomers of GFP-ST and GFP-SC are visible in the anti-GFP blot (arrows 5 and 6, respectively) along with bands of an unknown nature (marked “?”) which cannot be GFP bound to tHBcAg since these bands are not present on the anti-HBcAg blot. Blots cropped for clarity, for uncropped blots see Supplementary Fig. S4.

In order to demonstrate that the conjugation of GFP to the tandem core particles necessitates the interaction of SpyTag with SpyCatcher, GFP-ST or GFP-SC were co-expressed with unconjugated tandem core (tHBcAg) or tandem core conjugated to the same binding partner as the GFP moiety. Total soluble protein of the infiltrated leaf tissue was analysed by duplicate anti-HBcAg and anti-GFP western blots (Fig. 5). The best indicative sign of covalent interaction between GFP and tHBcAg is in the higher molecular weight multimers of tHBcAg constructs which are typically visible on a western blot of tandem core constructs produced in the cytosol of plant cells (labelled “1” in Fig. 5 but also visible in Fig. 3). This laddering/smearing effect is seen with all tandem core constructs in the anti-HBcAg blot but only for the tHBcAg-SC + GFP-ST combination in the anti-GFP blot (Fig. 5). There is no sign of covalent binding between GFP and tandem cores either in an incompatible interaction (with SpyTag conjugated to both moieties) or an impossible interaction (with GFP-ST or GFP-SC co-expressed with unconjugated tHBcAg). There is a band of about 56 kDa visible in the anti-GFP blot in samples where unconjugated tHBcAg is expressed (indicated by a question mark). While this could be a dimer of GFP-ST, its presence in the sample where GFP-SC was expressed is more mysterious. However it cannot be either form of GFP binding to unconjugated tHBcAg since this band is absent in the anti-HBcAg blot. Taken together, these data confirm that the covalent interactions seen in Figs. 3 and 4 are due to specific interaction between SpyTag and SpyCatcher.

### VLP assembly in the cytosol and ER and conjugation of tHBcAg and GFP

**Figure 6:**
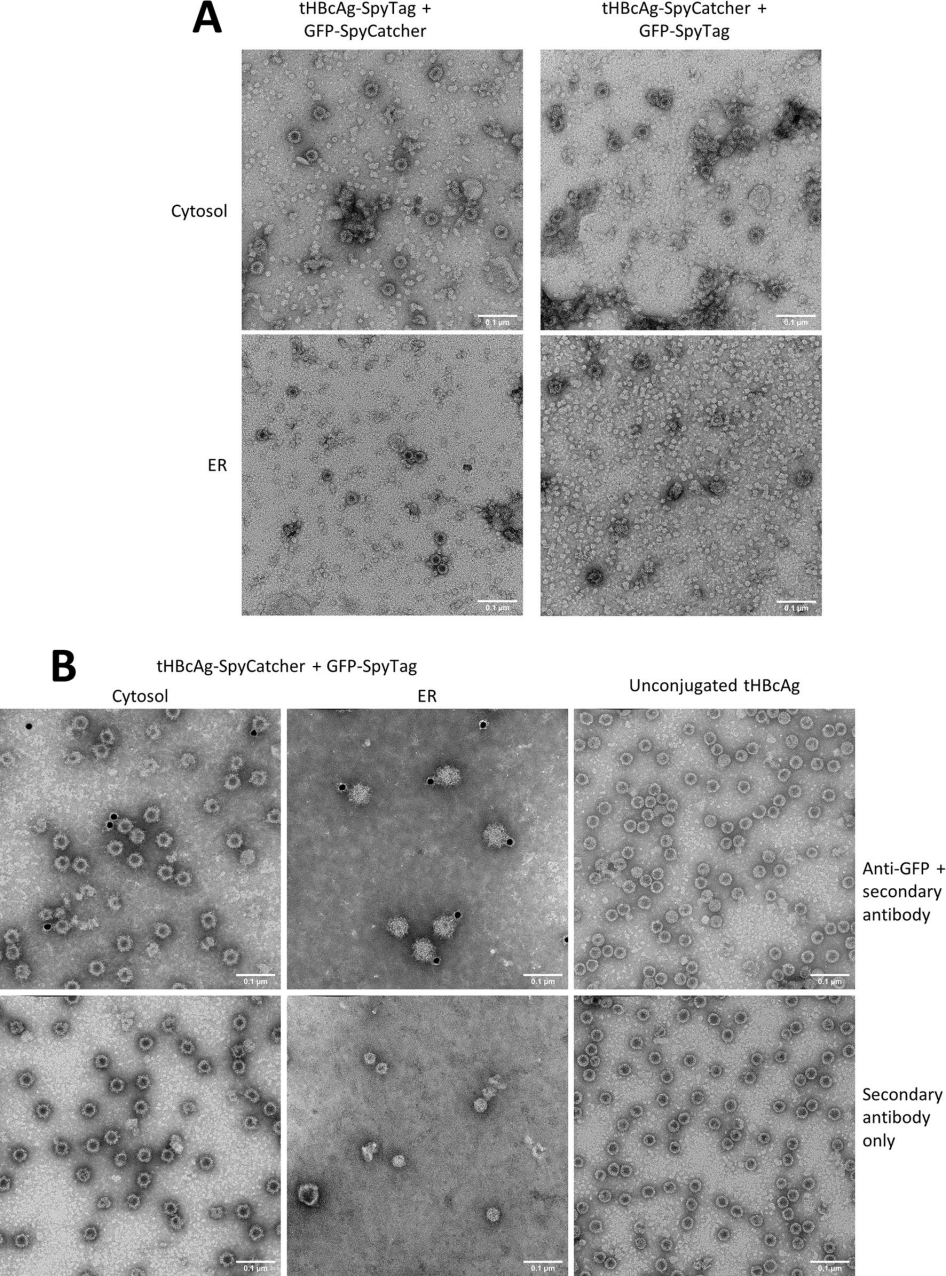
Transmission electron microscopy analysis of plant-produced VLPs. (**A**) 70% sucrose fractions. All constructs led to the assembly of tHBcAg VLPs in a comparable size and shape as previously published^28^. (**B**) Immunogold labelling of Nycodenz gradient-purified VLPs. Top row: grids incubated with anti-GFP primary antibody then gold-conjugated secondary antibody. Bottom row: grids incubated with gold-conjugated secondary antibody only. All samples were stained with 2% (w/v) uranyl acetate, scale bars are 100 nm each.

To verify that the 70% sucrose fractions indeed contain correctly assembled VLPs we analysed these fractions using transmission electron microscopy. Figure 6a shows that all tested combinations led to the detection of 25–30 nm HBcAg VLPs with a similar size and shape as described previously^28^. Moreover, VLPs produced from cytosolic and ER co-expression of tHBcAg-SC and GFP-ST were purified more thoroughly alongside unconjugated tHBcAg produced in the cytosol. These were purified by sucrose cushion followed by dialysis using a membrane with 100 kDa molecular weight cut-off (MWCO) to remove unbound GFP, then loaded onto a Nycodenz density gradient, using a previously published protocol^46^. The green fluorescent bands on the GFP-containing gradients were isolated (the equivalent fraction was isolated in the unconjugated tHBcAg gradient) and dialysed again using a 100 kDa MWCO membrane. Quantification of the final purified VLPs indicated a final purified yield of unconjugated tHBcAg and cytosolic tHBcAg-SC + GFP-ST of approximately 150 µg of purified VLPs per gram of fresh-weight infiltrated leaf tissue (FWT), whereas the ER-expressed tHBcAg-SC + GFP-ST had a final purified yield of approximately 6 µg/g FWT. These three preparations of particles were used for immunogold-labelling electron microscopy using an anti-GFP primary antibody and a secondary antibody conjugated to gold nanoparticles. This allowed detection of GFP on the surface of the VLPs conjugated to GFP (Fig. 6b).

### Conjugation of tHBcAg to HIV-P24

**Figure 7:**
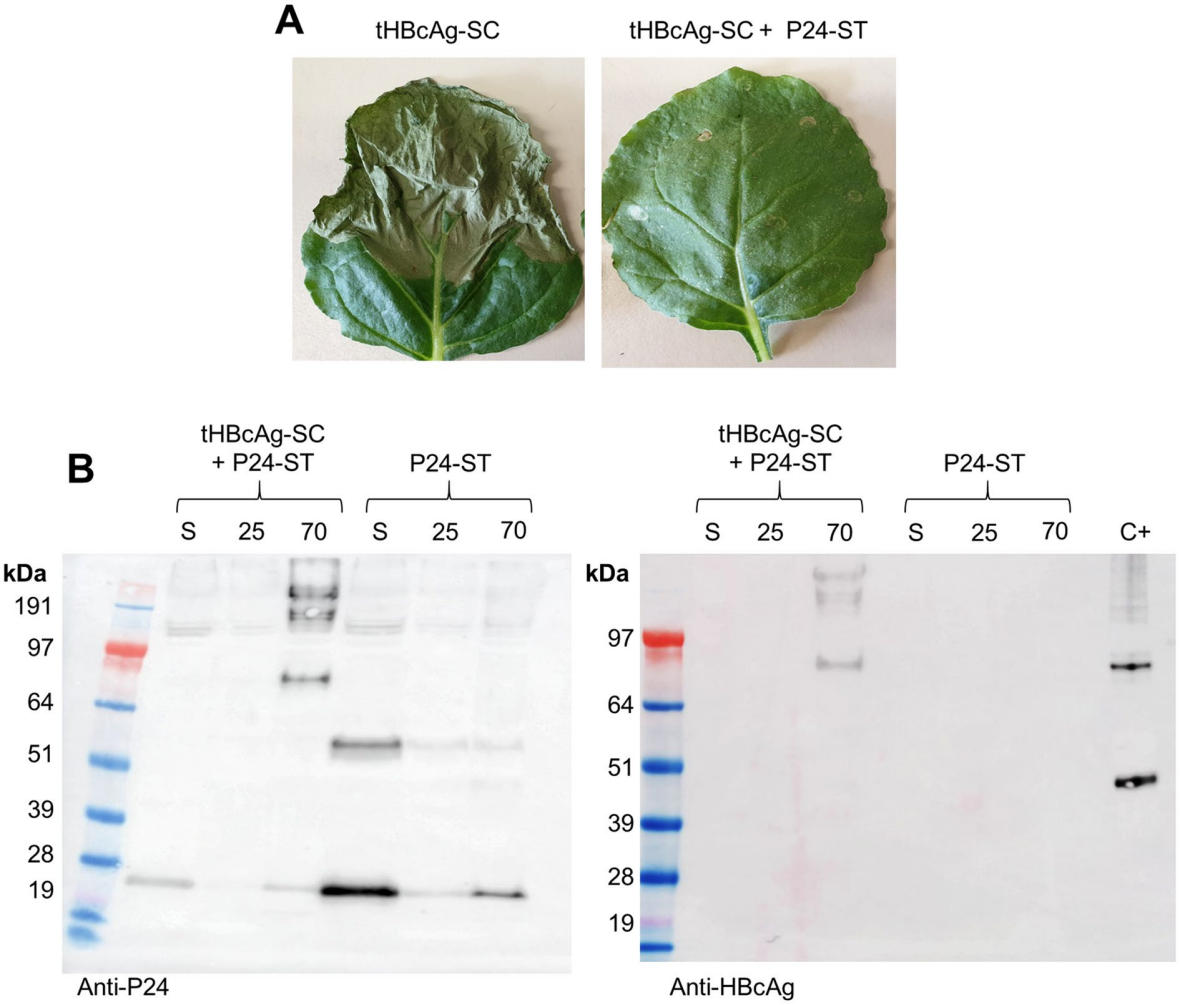
In vivo conjugation of tHBcAg-SC VLPs with P24-ST. (**A**) Phenotype of leaves infiltrated with tHBcAg-SC and tHBcAg-SC + P24-ST. Severe necrosis was caused by tHBcAg-SC as shown before. Co-expression of P24-ST substantially mitigated this effect indicating successful conjugation of VLPs and P24. (**B**) Duplicate Western blots of protein samples from leaves infiltrated with tHBcAg-SC + P24-ST or P24-ST alone (left: anti-P24, right: anti-HBcAg) after double sucrose cushion. Expected size of unconjugated P24 is 24 kDa, expected size after conjugation to tHBcAg is ~ 80 kDa. S = supernatant, 25 = 25% sucrose fraction, 70 = 70% sucrose fraction. C +  = tHBcAg-SC + GFP-ST as positive control. Blots cropped for clarity, for uncropped blots see Supplementary Fig. S5.

To verify whether the conjugation strategy described above works with proteins other than GFP, we conjugated tHBcAg VLPs with the HIV capsid protein, P24, by co-expressing tHBcAg-SC and P24-ST in the cytosol. The phenotype of infiltrated leaves (Fig. 7a) suggested that the conjugation was successful, because the severe phenotype observed with tHBcAg-SC alone was mitigated by the co-expression of P24-ST (Fig. 7a). This was verified by Western blot analysis (Fig. 7b). When P24-ST was expressed alone, it was detected in the expected monomeric size of about 24 kDa as well as multimeric sizes of about 50, 100 and 200 kDa in all three analysed fractions of the sucrose cushion, though it was most abundant in the supernatant. When tHBcAg-SC was co-expressed we observed P24- specific bands with a size of about 80 kDa in the 70% sucrose fraction, along with higher molecular weight specific bands (presumably multimers). This molecular weight corresponds to the conjugated proteins and presence of tHBcAg-SC in this fraction (and in bands of the same sizes) was also confirmed by Western blot (Fig. 7b). However, unconjugated P24-ST was detected in all three fractions as well, indicating an excess of P24 compared to tHBcAg. Interestingly, we could not detect any unbound tHBcAg-SC protein as observed in the tHBCAg-ST + GFP-SC control. This might be an artefact of western blot detection, but could also indicate complete occupancy of tHBcAg-SC with P24-ST.

## Discussion

The SpyTag/SpyCatcher system is a powerful tool for the conjugation of VLPs to peptides or whole proteins for vaccine design and therapeutic approaches. The conjugation reaction between the partners can take place in vitro by mixing purified proteins^33,47,48^, as well as in vivo when both partners are expressed in the same cell in *Escherichia coli*^33^. Furthermore, it was shown that conjugation partners can be expressed in separate plants and conjugation occurred during protein extraction by mixing the leaf material^40^. However, the separate expression of two proteins is more time- and material-consuming than co-expressing the conjugation partners within the same plant. Because the SpyTag/Catcher system is robust at different pH values, temperatures and buffer conditions^33^, we hypothesized that the conjugation reaction is possible in vivo in plants. Indeed, we showed for the first time that it is possible to conjugate tHBcAg VLPs with the model antigen GFP in either the cytosol or the ER of *Nicotiana benthamiana* and that the results of the cytosolic expression are transferrable to the HIV capsid protein P24. GFP has previously been used as a model antigen for conjugation studies with HBcAg VLPs^28,44^ and its fluorescence can be used as an indicator for correct protein folding^44^. We have shown that GFP remained active after conjugation and that is does not interfere with VLP formation in plants. These results are comparable to those obtained previously^28^ in which GFP was post-translationally linked to tHBcAg VLPs via a GFP-specific nanobody. However, the covalent nature of the SpyTag/SpyCatcher interaction, along with the easy fusion of SpyTag to a target antigen makes the system described here an overall improvement over the nanobody-based system.

We could detect differences in conjugation efficiency when we compared VLPs presenting SpyTag to VLPs displaying SpyCatcher. While Thrane et al.^47^ observed no differences for Acinetobacter phage AP205-derived VLPs, we show that it was more efficient to display the SpyCatcher in the MIR region of tHBcAg and the SpyTag on GFP, rather than vice-versa (although precise quantification of the absolute conjugation efficiencies are not currently within our means). The co-expression of tHBcAg-ST and GFP-SC led to lower conjugation efficiency than tHBcAg-SC and GFP-ST in the cytosol and barely any conjugated proteins could be detected when the former combination was targeted to the ER. We hypothesise that this may be due to greater avidity of SpyCatcher when it is displayed on the surface of a VLP rather than fused to an antigen. It is also possible that our observation is an effect of variations in stoichiometric concentrations of the various components, caused by differences in accumulation of the different constructs. Based on this observation and because the 10 amino acid SpyTag is less likely to interfere with antigen structure, the fusion of the SpyTag to the antigen and SpyCatcher to the VLP is likely to be the strategy of choice in future work. This is also supported by the observation of a negative correlation between antigen size and number of conjugated antigens^47^. However, Thrane et al.^47^ also described the possibility of improved antigen yield when fused to the SpyCatcher and our results are based on the study of a single partner protein. Consequently, we cannot draw a general conclusion and it is possible that testing both possible combinations for each new reaction might be necessary for optimal results.

Displaying the SpyCatcher on tHBcAg in the absence of a SpyTag-bound partner in the cytoplasm resulted in leaf necrosis. Because this effect is not visible with the ER-targeted version and is relieved by the presence of the SpyTag partner protein, we assume that non-specific conjugation to endogenous proteins is responsible for the severe phenotype. We found that the necrotic leaves would typically yield undetectable amounts of tHBcAg-SC VLPs. Hence, in vivo conjugation is not only advantageous but is in fact necessary with this system as opposed to mixing leaf material or in vitro conjugation of isolated proteins. However, no data for VLPs derived from other viruses displaying the SpyCatcher in plants are available to compare our result and the described effect might be specific for tHBcAg-SC VLPs.

While we could show that our strategy was successful and led to the conjugation of tHBcAg VLPs and the respective partners, we also observed unconjugated proteins in all groups. This was also observed when the SpyTag/SpyCatcher system was used to conjugate purified partners in vitro^32,33,47,48^ or during protein extraction^40^. Unconjugated GFP and P24 might indicate 100% occupancy of VLPs or steric hindrance on the VLP surface preventing any further conjugation. The presence of free VLPs and proteins might require additional purification steps to separate conjugated or unconjugated proteins. While we could determine that certain configurations result in greater conjugation efficiency than others, we did not have a means of reliably and accurately quantifying absolute conjugation efficiency. In any case, we hypothesise that this will depend on expression levels of the various binding partners as well as the size of the antigen, which may prevent 100% occupancy on the surface of the VLPs if it is too large.

In conclusion, this work presents an efficient strategy which can lead to time and cost saving post-translational, covalent conjugation of recombinant proteins in plants. It can be applied to cytosolic expression and we have shown the potential for directly conjugating ER- targeted proteins to tHBcAg VLPs in vivo in plants. Given that many target antigens are ER-targeted glycoproteins, we anticipate that future work is likely to focus on this promising avenue for novel vaccine design.

## Methods

### Design and cloning of expression vectors

The sequences of SpyTag (first 10 amino acids from PDB: 4MLI_B) and a truncated 86 aa version of SpyCatcher were amplified by PCR and cloned into the MIR of the C-terminal core of tHBcAg (pEAQ-t-EL described in^28^). The SpyTag and SpyCatcher sequences were fused to the *C*-terminus of GFP, amplified from pEAQ-*HT*-GFP^45^, resulting in tHBcAg-ST, tHBcAg-SC, GFP-ST and GFP-SC.

For protein targeting and retention in the ER we added a *C*-terminal KDEL sequence and a *N*-terminal, 22 amino acid transit peptide from *Arabidopsis thaliana* basic chitinase (MKTNLFLFLIFSLLLSLSSAEF)^49^ to tHBcAg-ST/SC and GFP-ST/SC resulting in tHBcAg-ST/SC-ER and GFP-ST/SC-ER.

The sequence of HIV-P24 (GenBank AAB50258) was codon optimized for *Nicotiana benthamiana* and synthesised by GeneArt (Life Technologies Ltd.). The *C*-terminal SpyTag was added by overlapping extension PCR.

All constructs were cloned into pEAQ-*HT*^45^ using AgeI/XhoI restriction sites. Plasmids were transformed into *E. coli* Top10 cells (Life Technologies Ltd, Thermo Fisher Scientific, UK) for propagation and sequences were confirmed by sanger sequencing. All plasmids were then transformed into *Agrobacterium tumefaciens* LBA4404 for transient expression in *Nicotiana benthamiana*.

### Transient expression in *N. benthamiana*

Plants were grown and infiltrated as previously described^50^. In brief, LBA4404 cultures were adjusted to a final OD_600_ of 0.3–0.4 in MMA medium (10 mM MES buffer, pH 5.6; 10 mM magnesium chloride; 100 µM acetosyringone). *Nicotiana benthamiana* plants were infiltrated three weeks after pricking out using a needleless syringe.

### Protein extraction, demonstration of conjugation and isolation of virus-like particles

Plants were harvested at 7 dpi and, if GFP expression was being monitored, imaged under UV and white light. Protein was extracted from leaf material as described previously^46^. Briefly, infiltrated leaf material was weighed and blended in three volumes of sodium phosphate buffer (SPB) (0.1 M sodium phosphate, pH 7.2, supplemented with cOmplete protease inhibitor cocktail tablets (Roche, Grenzacherstrasse 124 CH-4070 Basel,Switzerland). Crude extract was filtered through one layer of Miracloth (MerckMillipore) and clarified by centrifugation (15,000×*g*, 4 °C, 20 min). Supernatant was filtered through 0.45 µm syringe filters (Merck Millipore, Sartorius) and loaded on a 25% (w/v) (5 ml) and 70% (w/v) (1 ml) double-layer sucrose cushion prepared in 0.1 M SPB, followed by centrifugation for 3 h and 4 °C at 167,000×*g* (30,000 rpm in a SureSpin 360/36 rotor, Thermo Fisher Scientific). For visualisation of GFP, ultracentrifuge gradients were imaged under UV light directly after centrifugation. Fractions were collected from the bottom of the tube (2 ml 70% sucrose and interface to 25% sucrose, 2 ml 25% sucrose and 2 ml of the supernatant). For quantification and immunogold labelling, VLPs were further purified according to a previously published protocol^46^. After initial concentration on a double-layered sucrose cushion, the 70% and interface layers were dialysed against 20 mM ammonium bicarbonate in Float-a-Lyzer dialysis cassettes with a 100 kDa molecular weight cut-off (MWCO, Spectrum Laboratories Inc.). The recovered dialysates were clarified by centrifugation for 20 min at 16,000×*g* then concentrated on a vacuum concentrator (SpeedVac) to a final volume of about 1.5 ml then loaded onto a Nycodenz density gradient composed of equal volume layers of 20, 30, 40, 50 and 60% (w/v) Nycodenz in PBS. After ultracentrifugation at 273,800×*g* (40,000 rpm in a TH641 rotor, Sorvall) for 3 h at 4 °C, gradients were fractionated in 0.5 ml fractions. Anti-HBcAg western blotting was used to identify HBcAg-containing fractions and the fractions with the strongest signal in the western blot (which included the fractions that fluoresced green for the gradients containing GFP) were pooled, dialysed in phosphate buffered saline (PBS) using 100 kDa MWCO Float-a-Lyzer dialysis cassettes, clarified by centrifugation for 20 min at 16,000×*g* then filtered over a 0.45 µm syringe filter. Quantification of the VLPs was carried out using the BCA protein assay kit (Pierce) using a bovine serum albumin (BSA) standard curve.

### Western blot analysis

Samples were analysed by electrophoresis on 4–12% (w/v) NuPAGE Bis–Tris gels (Novex, Life Ttechnoloiges, USA) and proteins were blotted on a nitrocellulose membrane. Membranes were blocked overnight in blocking buffer (5% (w/v) milk in PBS with 0.1% (v/v) Tween 20). Primary antibodies were added for 2 h at room temperature in blocking buffer as follows: mouse anti-HBcAg (10E11, Abcam ab8639) 1:4000. Mouse anti-GFP (horseradish peroxidase (HRP)-conjugated, Abcam ab190584) 1:7000. Mouse anti-P24 (HRP conjugated, Abcam ab53841) 1:2000. For tHBcAg Western goat anti-mouse (ThermoFisher scientific, 62–6520) was used in a 1:10.000 dilution in blocking buffer. For development, chemiluminescent substrate (Immobilon, Millipore, USA) was used and chemiluminescence was detected using an ImageQuant LAS 500 (GE Healthcare UK Ltd., United Kingdom).

### Negative-stain transmission electron microscopy (TEM) and immunogold labelling

Samples were adsorbed on a 400 mesh carbon-coated copper grid (EM resolutions UK) for 30 s. The grids were washed with 8 drops of H_2_O_dd_ and stained with 2% (w/v) uranyl acetate for 15–30 s and imaged using a Talos F200C TEM fitted with a Gatan OneView camera. For immunogold labelling, VLPs were adsorbed onto grids which were then washed with PBS then blocked with blocking solution (1% (w/v) BSA and 0.1% (v/v) Tween-20 in PBS) for 30 min before equilibration in equilibration solution (0.1% (w/v) BSA and 0.1% (v/v) Tween-20 in PBS) for 1 min followed by incubation with the primary antibody (rabbit polyclonal to GFP, Abcam ab6556) diluted 1:100 in equilibration solution for 1 h. After washing the grids in equilibration solution (three 5-min washes), the secondary antibody (goat anti-rabbit conjugated to gold nanoparticles, BBI Solutions EM.GAR15) diluted 1:40 in equilibration solution was incubated on the grid for 1 h before washing in wash solution (0.01% (v/v) Tween-20 in PBS; four 5-min washes) then PBS (two 2-min washes) then water (three 3-min washes) followed by negative staining with 2% (w/v) uranyl acetate. Controls were produced by incubating with the secondary antibody only to ensure that signal was due to primary antibody binding and not background from the secondary antibody.

## Supplementary information

Supplementary Information.

## Data Availability

No large datasets were generated during the course of this study. All data relevant to this study will be made available upon reasonable request addressed to the corresponding author.
