# Supplementary material for: Covalent protein display on Hepatitis B core-like particles in plants through the in vivo use of the SpyTag/SpyCatcher system
Source: Sci Rep. 2020 Oct 13;10:17095. doi: 10.1038/s41598-020-74105-w (PMC7555512; doi:10.1038/s41598-020-74105-w)
Supplement: Supplementary file 1 — Supplementary Information. [file 41598_2020_74105_MOESM1_ESM.pdf]

Supplementary Information

Covalent protein display on Hepatitis B core-like particles in plants through the *in vivo* use of the SpyTag/SpyCatcher system.

Hadrien Peyret[1*], Daniel Ponndorf[1], Yulia Meshcheriakova[1] Jake Richardson[2], and George P. Lomonossoff[1]

1: Department of Biological Chemistry, John Innes Centre, NR4 7UH UK

2: Department of Cell and Developmental Biology, John Innes Centre, NR4 7UH, UK

* Corresponding author

Hadrien.peyret@jic.ac.uk*

Daniel.ponndorf@jic.ac.uk

Yulia.meshcheriakova@jic.ac.uk

Jake.richardson@jic.ac.uk

George.lomonossoff@jic.ac.uk

Keywords: SpyTag, SpyCatcher, *N. benthamiana*, Virus-like particles, *in vivo* conjugation, tandem core, Hepatitis B core antigen, plant molecular farming, GFP, P24, antigen display

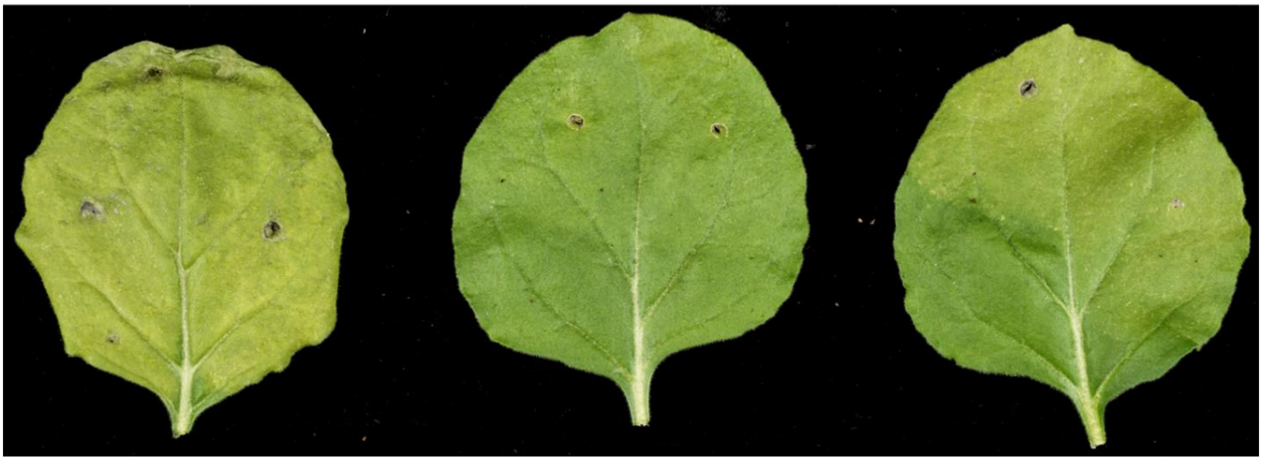

Supplementary Figure S1: *Symptoms on agroinfiltrated leaves harvested 7 days post-infiltration. While leaves expressing untagged tHBcAg show chlorosis, leaves expressing GFP conjugated to SpyTag (GFP-ST) or SpyCatcher (GFP-SC) exhibit little to no chlorosis.*

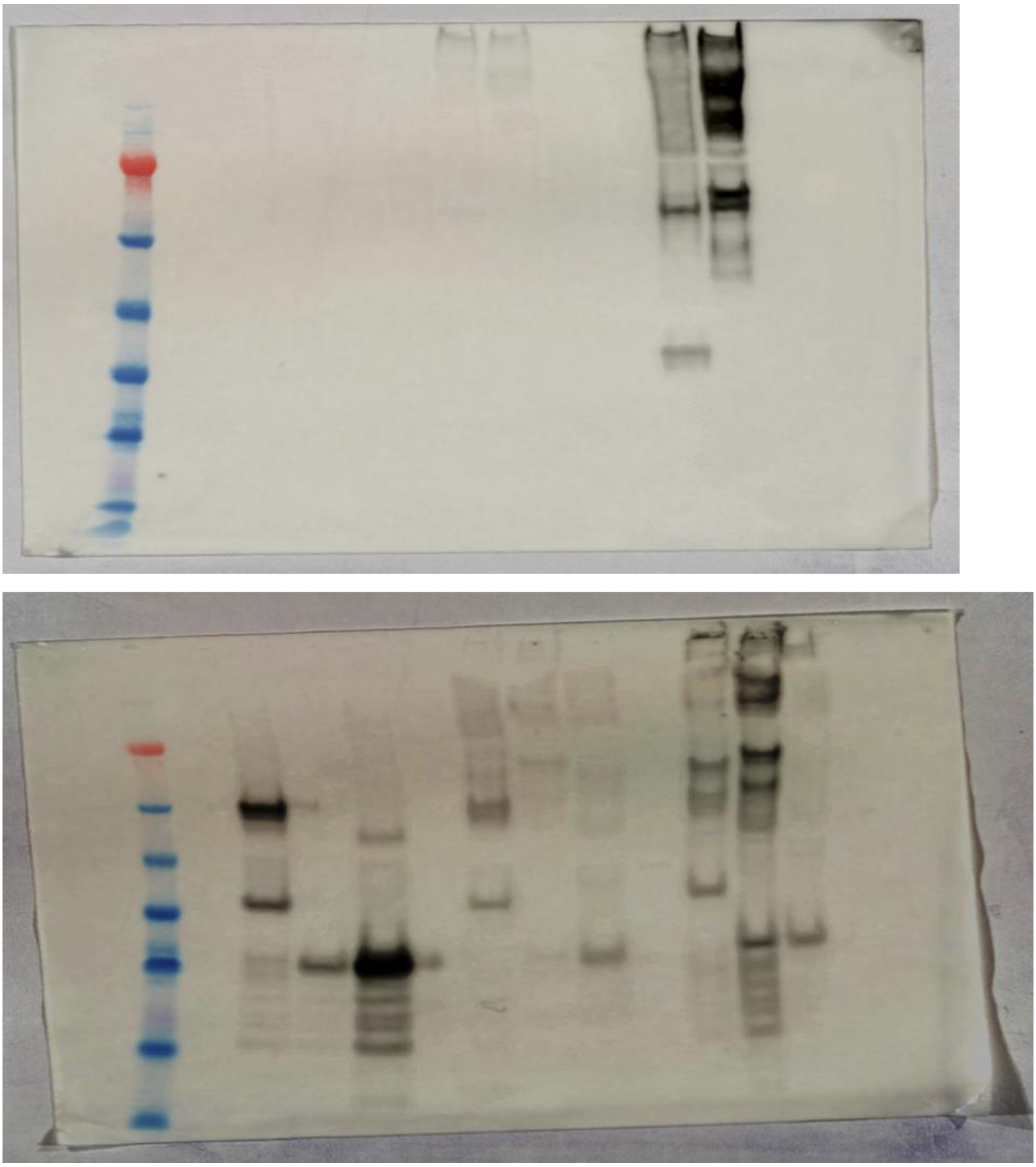

Supplementary Figure S2: *Uncropped western blots from Figure 3. Top: anti-HBcAg blot. Bottom: anti-GFP blot. Images obtained from ImageQuant LAS 500 (GE Healthcare UK Ltd., United Kingdom).*

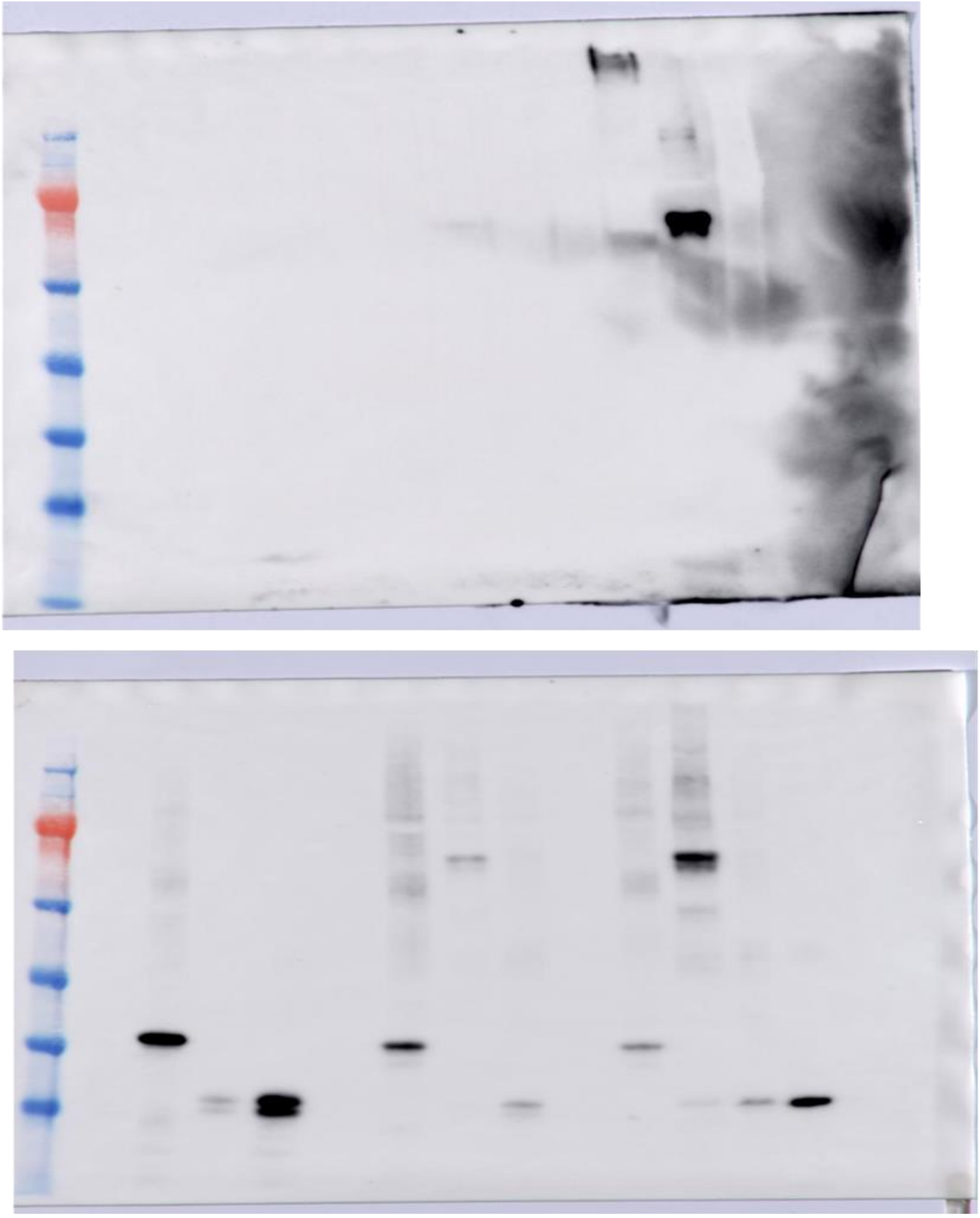

Supplementary Figure S3: *Uncropped western blots from Figure 4. Top: anti-HBcAg blot. Bottom: anti-GFP blot. Images obtained from ImageQuant LAS 500 (GE Healthcare UK Ltd., United Kingdom).*

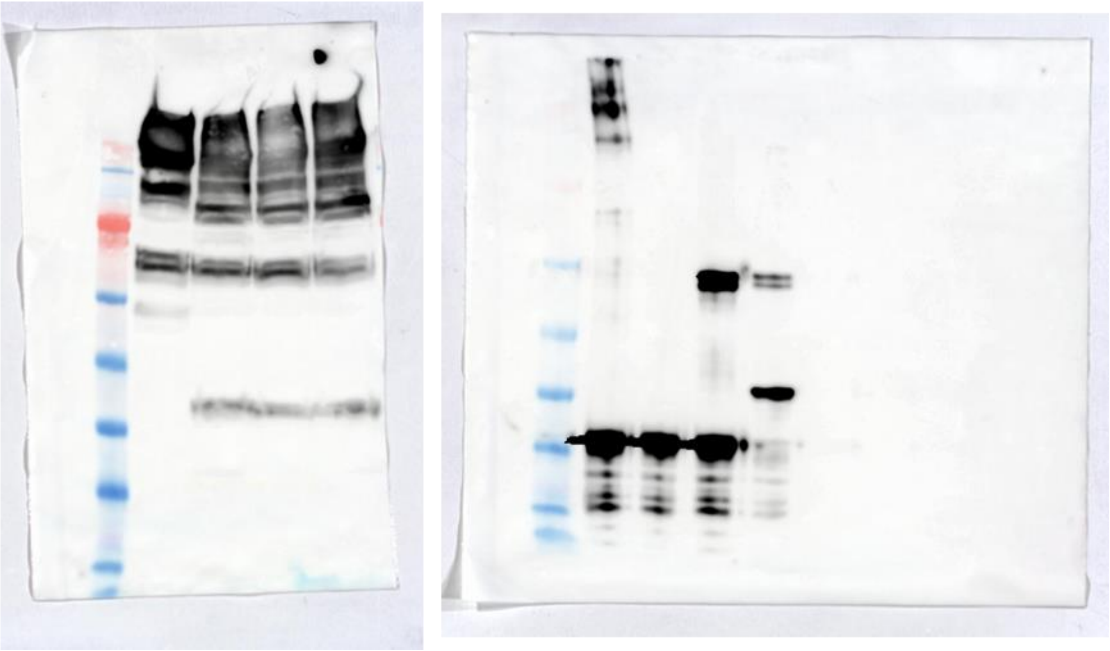

Supplementary Figure S4: *Uncropped western blots from Figure 5. Left: anti-HBcAg blot. Right: anti-GFP blot. Images obtained from ImageQuant LAS 500 (GE Healthcare UK Ltd., United Kingdom).*

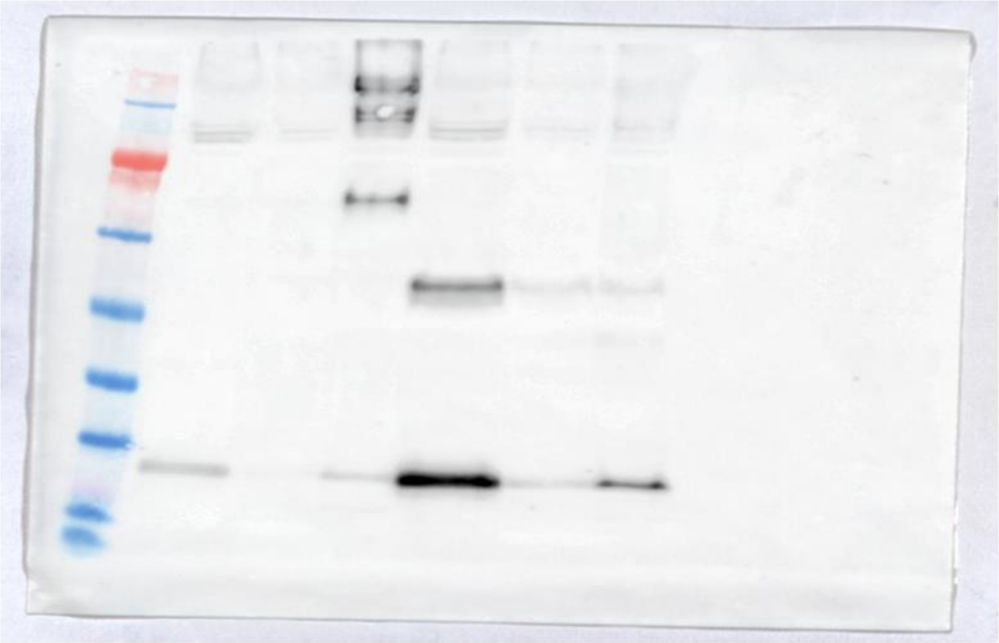

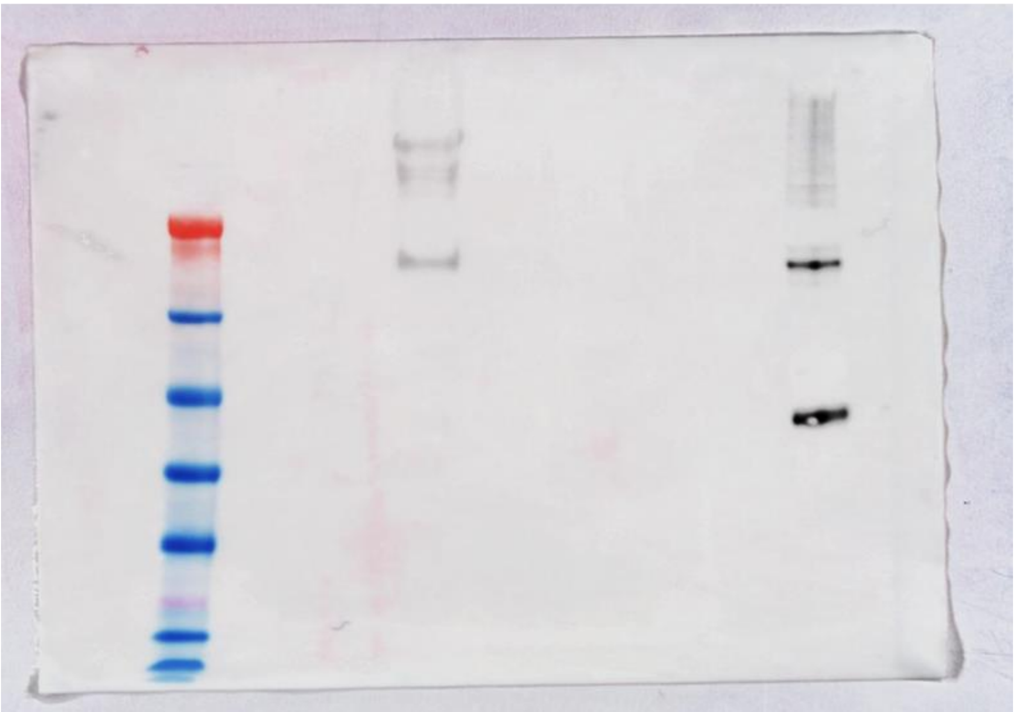

Supplementary Figure S5: *Uncropped western blots from Figure 7. Top: anti-P24 blot. Bottom: anti-HBcAg blot. Images obtained from ImageQuant LAS 500 (GE Healthcare UK Ltd., United Kingdom).*
